# A Minimally Invasive, Extracellular Vesicle-Based Approach for Monitoring Measurable Residual Disease in Acute Myeloid Leukemia: A Proof-of-Concept Study

**DOI:** 10.3390/cells15121068

**Published:** 2026-06-11

**Authors:** Helena Branco, Joana Carreira, Inês Soure, Cristina P. R. Xavier, Andreia Rosário, Maria Amorim, Hugo Osório, José E. Guimarães, Ana Bela Sarmento-Ribeiro, Manuel A. Sobrinho-Simões, Hugo R. Caires, M. Helena Vasconcelos

**Affiliations:** 1 i3S—Instituto de Investigação e Inovação em Saúde, Universidade do Porto, Rua Alfredo Allen 208, 4200-135 Porto, Portugal; hbranco@ipatimup.pt (H.B.); jscarreira@ualg.pt (J.C.); ines.msoure@gmail.com (I.S.); andreia_rosario11@hotmail.com (A.R.); hosorio@i3s.up.pt (H.O.); jeteguimaraes@gmail.com (J.E.G.); manuel.simoes@chsj.min-saude.pt (M.A.S.-S.); hugo.caires@live.com.pt (H.R.C.); 2 Cancer Drug Resistance Group, IPATIMUP—Institute of Molecular Pathology and Immunology, University of Porto, Rua Júlio Amaral de Carvalho 45, 4200-135 Porto, Portugal; 3 Laboratório de Oncobiologia e Hematologia, Clinica Universitária de Hematologia, FMUC—Faculty of Medicine, University of Coimbra, Azinhaga de Santa Comba, 3000-548 Coimbra, Portugal; ana.belasarmento@gmail.com; 4 Department of Chemistry, University of Aveiro, Campus Universitário de Santiago, 3810-193 Aveiro, Portugal; 5 Associate Laboratory i4HB—Institute for Health and Bioeconomy, University Institute of Health Sciences—CESPU, 4585-116 Gandra, Portugal; 6 UCIBIO—Applied Molecular Biosciences Unit, Toxicologic Pathology Research Laboratory, University Institute of Health Sciences (1H-TOXRUN, IUCS-CESPU), 4585-116 Gandra, Portugal; 7 Serviço de Hematologia Clínica, Centro Hospitalar Universitário de São João, Alameda Prof. Hernâni Monteiro, 4200-319 Porto, Portugal; amorim.ml@gmail.com; 8 FMUP—Faculty of Medicine, University of Porto, Alameda Prof. Hernâni Monteiro, 4200-319 Porto, Portugal; 9 Coimbra Institute for Clinical and Biomedical Research (iCBR)—Grupo de Ambiente, Genética e Oncobiologia (CIMAGO) and Center for Innovative Biomedicine and Biotechnology (CIBB), University of Coimbra, Azinhaga Santa Comba, 3000-548 Coimbra, Portugal; 10 Serviço de Hematologia Clínica, Centro Hospitalar e Universitário de Coimbra (CHUC), Praceta Professor Mota Pinto, 3000-561 Coimbra, Portugal; 11 Department of Biological Sciences, FFUP—Faculty of Pharmacy, University of Porto, Rua de Jorge Viterbo Ferreira 228, 4050-313 Porto, Portugal

**Keywords:** acute myeloid leukemia, measurable residual disease, liquid biopsies, extracellular vesicles

## Abstract

Measurable residual disease (MRD) in acute myeloid leukemia (AML) is monitored through detection of leukemia-associated phenotypic protein markers (LAPMs) in bone marrow aspirates, hindering disease real-time monitoring. We explored peripheral blood (PB), extracellular vesicle (EV)-based methods for MRD monitoring. To confirm that LAPMs are present in AML-derived EVs, EVs were isolated from OCI-AML3 cells by differential centrifugation and characterized according to their size (nanoparticle tracking analysis), morphology (transmission electron microscopy) and protein cargo (proteomic analysis and Western blot). CD14 and CD33 were detected in OCI-AML3 cells and their released EVs. To select a method to isolate EVs from the PB of AML patients, three techniques were tested: size exclusion chromatography followed by ultrafiltration (SEC-UF), Total Exosome Isolation Kit (Invitrogen) and Exo-spin™ Exosome Purification Kit (Cell Guidance Systems). SEC-UF allowed EV isolation with higher purity and less aggregates than the other techniques. LAPMs were detected in those EVs, but their presence depended on the isolation method. Finally, EVs from seven AML patients’ plasma were isolated by SEC-UF. LAPMs were identified in paired samples at diagnosis and remission, with differential expression throughout disease evolution. This proof-of-concept study highlights the possibility of real-time MRD monitoring through LAPMs’ analysis in AML patient’s circulating EVs.

## 1. Introduction

Acute myeloid leukemia (AML) is a malignant hematopoietic stem cell disorder characterized by the clonal expansion of undifferentiated myeloid progenitor cells in the bone marrow (BM) and peripheral blood (PB), severely affecting the normal production of differentiated blood cellular components [1,2]. AML is the most common acute leukemia in adults but constitutes only 12% of hematopoietic malignancies; it is one of the most life-threatening hematological cancers, with a 5-year survival rate below 15% in elderly patients and of 30–40% in patients under the age of 60 [3,4,5]. Indeed, although response rates to induction chemotherapy are considerable, with 40–60% of older patients and 60–80% of younger patients achieving a morphological complete remission, the presence of morphologically undetectable residual amounts of leukemia—commonly referred to as measurable (formerly minimal) residual disease (MRD)—ultimately translates into a high frequency of post-treatment relapse and morbidity [6,7,8].

Over the past few decades, MRD has been identified as a valuable post-treatment prognostic indicator that can aid in patient risk stratification and therapeutic management [8,9]. In fact, in patients with chronic myeloid leukemia, acute lymphoblastic leukemia or acute promyelocytic leukemia, MRD monitoring is already integrated into daily clinical practice [8,10,11,12]. Nevertheless, for the remaining AML sub-entities, MRD monitoring is still a field under exploration.

In AML, leukemic blasts frequently display aberrant immunophenotypes resulting from cross-lineage antigen expression, abnormal antigen expression levels, or asynchronous expression of immature and mature markers [13]. These leukemia-associated phenotypic protein markers (LAPMs) are identified in up to 90% of AML patients and present the basis for the detection of MRD [14]. Cross-lineage expression is characterized by the presence of lymphoid or NK-cell markers, such as CD2, CD3, CD5, CD19, or CD56, on myeloid blasts, whereas asynchronous expression involves the co-expression of immature antigens (e.g., CD34 or CD117) with markers of myeloid maturation (e.g., CD11c, CD14, CD15, or CD65). In addition, aberrant overexpression or underexpression of normally present antigens, including CD34, CD123, and HLA-DR, further contributes to the unique immunophenotypic profiles exploited for sensitive MRD monitoring [14,15,16].

Although peripheral blood liquid biopsies have been investigated for over a decade, their current application in a clinical setting remains limited. Conventional MRD monitoring is performed through two main methodologies, multi-parameter flow cytometry and real-time quantitative polymerase chain reaction. For both approaches, different LAPMs are now clinically described and useful to detect MRD [9,17]. However, these techniques are still not fully standardized or universally applicable, and, although PB-based analyses have been explored in limited settings, they often rely on invasive BM aspirates, which, despite offering increased sensitivity and specificity, also present several drawbacks [18]. Indeed, the invasive and traumatic nature of BM sampling severely hinders the real-time monitoring of the disease and only allows the collection from a single tumor site, which may not be representative of the tumor’s heterogeneity and does not take into consideration the occurrence of extramedullary disease [19,20]. Therefore, recent interest has been focused on the so-called PB-based liquid biopsies, since they would allow minimally invasive disease detection and real-time monitoring of the disease and, additionally, would be more cost-effective and simpler to perform [21]. Indeed, a robust concordance between the MRD levels detected in BM and PB has been described, suggesting that measuring MRD in PB rather than in BM aspirates may be a suitable strategy for MRD monitoring in the future [22]. Furthermore, evidence suggests that MRD monitoring via PB sampling may yield more representative and informative results than MRD monitoring through BM aspirates, reflecting the actual disease status and burden [23,24].

Recently, extracellular vesicles (EVs) have been recognized as a potential source of cancer biomarkers [25,26]. EVs are cell-released nanosized particles (30–5000 nm), enclosed by a lipid bilayer, which do not contain a functional nucleus and therefore cannot replicate [27,28]. These small particles present a wide range of biological features that make them attractive sources of disease-related biomarkers [29]. In fact, EVs are released into the extracellular space by nearly all cell types within the body and can be recovered from a wide range of biological fluids [30,31,32,33,34]. Furthermore, the lipid bilayer that surrounds EVs protects their cargo from external enzymatic degradation, making them a more stable and reliable source of disease biomarkers when compared to circulating free molecules [26,35]. Importantly, evidence suggests that EVs’ cargo derives from their cells of origin [25,26]. In AML, in particular, EVs have been demonstrated to play fundamental roles in tumorigenesis, cell proliferation, survival, angiogenesis and chemotherapy resistance, and, interestingly, newly diagnosed AML patients were demonstrated to present elevated levels of EVs in their plasma, when compared to healthy individuals [36,37,38,39,40]. Remarkably, a decrease in EVs levels has been described following treatment response in both pediatric and adult AML patients [41,42]. Thus, the development of alternative PB EV-based methods could be of great potential.

Here, we sought to determine whether AML-derived EVs could have the potential to be a source of AML disease biomarkers, as a proof-of-concept study to explore the possible application of a PB EV-based liquid biopsy to detect MRD biomarkers in AML patients. Thus, our proof-of-concept work focused on three very specific aims: (1) verify the presence of LAPMs in the cargo of EVs shed by a human AML cell line; (2) select an appropriate method for EV isolation from the PB of AML patients; and (3) analyze LAPMs present in AML patients’ plasmatic EVs and the possibility of correlating their levels with disease progression.

## 2. Materials and Methods

### 2.1. Cell Culture

The OCI-AML3 cell line was obtained as a kind gift from Dr. D. Duarte, i3S, Porto, Portugal and used for EV isolation and characterization. The HL60 cell line was purchased from the American Type Culture Collection (ATCC), and the KG1a cell line was a kind gift from Dr. L. Martins, MRC, UK (originally from ATCC); their cell lysates were used as positive controls on Western blots. The OCI-AML3 and HL60 cell lines were maintained in Roswell Park Memorial Institute (RPMI) 1640 medium supplemented with Stable Glutamine and 25 mM HEPES (Biowest, Nuaillé, L0496-500), supplemented with 20% or 10% fetal bovine serum (FBS), respectively. The KG1a cell line was maintained in Dulbecco’s Modified Eagle Medium (DMEM) supplemented with 4.5 g/L Glucose with UltraGlutamine^TM^ w/sodium pyruvate (Lonza, Basel Stücki, Switzerland; BE12-604F), enriched with 10% fetal bovine serum (FBS; Biowest, Nuaillé, France; S181H-500). Cells were incubated at 37 °C in a humidified chamber containing 5% CO_2_. For EV isolation, medium was supplemented with 20% EV-depleted FBS (centrifuged at 100,000 *g*, 4 °C, for at least 16 h), and cells were seeded at 1 × 10^6^ cells/mL, corresponding to a total of 1.82 × 10^8^ cells. Cells were genotyped and routinely tested for possible mycoplasma infection (Genomics and Cell Culture and Genotyping Services, i3S, Porto, Portugal). All experiments were carried out with cells at the exponential growth phase and with more than 90% viability.

### 2.2. Cell Lysis

Cell pellets were obtained following centrifugation of the conditioned medium at 300 *g* for 10 min at RT and washed twice with phosphate buffered saline 1× (PBS; Merck Life Science, Darmstadt, Germany; P5493) by centrifugation at 1200 rpm for 5 min at 4 °C (Centrifuge 5810; Eppendorf, Hamburg, Germany). The obtained pellet was again resuspended in 1 mL of PBS 1×, transferred to a 1.5 mL tube and centrifuged at 1000 rpm for 5 min at 4 °C (MicroStar 17R Refrigerated Microcentrifuge; VWR, Lutterworth, UK). The acquired cell pellet was then lysed in Wynman’s buffer [1% NP-40 (Merck Life Science, Darmstadt, Germany; 74385), 0.1 M Tris-HCl pH 8.0 (Merck Life Science, Darmstadt, Germany; T-7149), 0.15 M NaCl (Merck Life Science, Darmstadt, Germany; S3014), 5 mM EDTA (Merck Life Science, Darmstadt, Germany; E0399)] complemented with a protease inhibitor cocktail (Roche, Basel, Switzerland; 11836145001) and phosphatase inhibitor (Merck Life Science, Darmstadt, Germany; S6508), for 30 min at 4 °C. Protein lysates were obtained after centrifugation at 13,000 rpm for 10 min at 4 °C.

### 2.3. Selection and Processing of Human Blood Samples

#### 2.3.1. Blood Collection

PB samples were collected from AML patients from “Centro Hospitalar Universitário de São João” (CHUSJ, Porto, Portugal) in 3.8% sodium citrate (0.129 M) tubes (Vacutest^®^; KIMA, Padova, Italy). Paired PB samples were collected from patients at diagnosis and after induction chemotherapy had produced a morphological complete remission. In three patients a relapse sample was also obtained. The study was approved by the Ethics Committee of CHUSJ, Porto, Portugal. Informed consent was obtained from all participants in accordance with the Declaration of Helsinki.

#### 2.3.2. Clinical Information

Clinical information was collected after patient anonymization through the generation of a unique patient number.

#### 2.3.3. Poor Platelet Plasma Isolation

PB samples were maintained at RT in an orbital shaker with gentle agitation (50 rpm) until poor platelet plasma (PPP) isolation. In order to isolate PPP, the blood samples were transferred, in sterile conditions, to a 15 mL centrifuge tube and centrifuged at 2500 *g* (Megafuge 1.0R; Heraeus, Hanau, Germany) for 15 min at 18–20 °C. The platelet-rich plasma layer was transferred to another 15 mL centrifuge tube and centrifuged again at the same previously described conditions. The supernatant containing PPP was collected to another 15 mL centrifuge tube and mixed with an equal volume of sterile 0.32% (*w*/*v*) trisodium citrate dihydrate (Merck Life Science, Darmstadt, Germany; S1804) in PBS [0.137 M NaCl (Merck Life Science, Darmstadt, Germany; S3014), 0.0027 M KCl (Merck Life Science, Darmstadt, Germany; P-9541), 0.01 M Na_2_HPO_4_.H_2_O (Merck Life Science, Darmstadt, Germany; K19368380) and 0.0018 M KH_2_PO_4_ (Merck Life Science, Darmstadt, Germany; A591973)] (pH 7.4, 0.22 µm filtered). Aliquots of 1 mL of PPP were stored in 1.5 mL tubes and frozen at −80 °C until used.

### 2.4. EV Isolation Methods

#### 2.4.1. Ultracentrifugation

The OCI-AML3 cell line was cultured in medium supplemented with 20% EV-depleted FBS during 72 h. Then, EVs were isolated by differential centrifugation, following a protocol previously described by Théry et al. (2006), with some modifications [43]. First, the conditioned medium was transferred to sterile 50 mL centrifuge tubes and centrifuged at 300 *g* for 10 min at room temperature (RT) (Centrifuge 5810; Eppendorf, Hamburg, Germany) to collect the cell pellet. Then, the supernatant was transferred to a new 50 mL centrifuge tube and centrifuged at 2000 *g* for 30 min at 4 °C (Centrifuge 5810; Eppendorf, Hamburg, Germany) to eliminate apoptotic bodies and cell debris. Subsequently, the obtained supernatant was transferred to Quick-Seal Polycarbonate tubes (Beckman Coulter, Brea, CA, USA; 355618) and subjected to an ultracentrifugation at 100,000 *g* for 75 min at 4 °C (Ultracentrifuge Beckman Optima XPN 100; Beckman Coulter, CA, USA) using the type 70 Ti fixed-angle titanium rotor (Beckman Coulter, Brea, CA, USA; 337922). After that time, the supernatant was discarded, and the EV pellets were washed with sterile, filtered 0.32% Sodium Citrate in PBS (H 7.4, 0.22 µm filtered) to eliminate contaminating proteins from the medium. Then, EVs were transferred to a new Quick-Seal Polycarbonate tube, and a second ultracentrifugation was then performed under the previously described conditions. The obtained EV-containing pellet was further resuspended in 100–150 μL of 0.32% Sodium Citrate in PBS (pH 7.4, 0.22 µm filtered) and either used immediately for EV characterization or stored at −80 °C until further use.

#### 2.4.2. Size Exclusion Chromatography (SEC)

EV isolation through size exclusion chromatography (SEC) was performed using a method previously described by Anita Boing et al. (2014), with some modifications [44]. Briefly, approximately 22 mL of Sepharose CL-2B (Merck Life Science, Darmstadt, Germany; CL2B300) were washed with 0.32% Sodium Citrate in PBS (pH 7.4, 0.22 µm filtered) and dropped on a 10 mL syringe (SOFT-JECT^®^; Henke Sass Wolf, Tuttlingen, Germany; 5100-X00V0) filled with a nylon stocking at the tip. After reaching the 10 mL mark and overnight compacting, a paper filter (3 MM CHR; Cytiva, Marlborough, MA, USA; 3030-917) was added to the top of the compacted Sepharose to create an even stacking interface. Before storage, the column was washed with 20% ethanol solution (bacteriostatic agent) and then submersed in this solution within a 50 mL falcon and stored at 4 °C until further use. Before EV isolation, the SEC column was first washed with 0.32% Sodium Citrate in PBS (eluent) to remove the bacteriostatic agent. Then, and before the eluent in the column dried, 0.9–1.0 mL of PPP was loaded into the column and allowed to enter within the Sepharose component before adding continuously more eluent. A total of 15 fractions with 1 mL each were collected in 1.5 mL tubes.

#### 2.4.3. Ultrafiltration

Briefly, fractions 3 to 6 obtained from the SEC were added to the top of a spin filter of 100 kDa cut-off membrane (Amicon^®^ Ultra-4 Centrifugal Filters Ultracel^®^—100K; Merck Life Science, Darmstadt, Germany; UCF8100) and centrifuged at 4000 rpm for approximately 60 min at 4 °C. The concentrated sample was then collected, quantified (please see Section 2.5.1 and Section 2.5.2) and either used immediately for EV characterization or stored at −80 °C until further use.

#### 2.4.4. Total Exosome Isolation Kit (From Plasma)

EVs were isolated from plasma using the commercially available Total Exosome Isolation Kit (Thermo Fisher Scientific, Sunnyvale, CA, USA), following the manufacturer’s instructions. First, the PPP sample was subjected to a centrifugation at 2000 *g* for 20 min at RT, which was followed by a second centrifugation at 10,000 *g* for 20 min at RT. Then, the clarified PPP was transferred into a new tube and mixed with 0.5 volumes of PBS and 0.2 volumes of the Exosome Precipitation Reagent (from plasma), provided in the kit. Following a 10 min incubation period, the sample was centrifuged at 10,000 *g* for 5 min at RT, and the supernatant was discarded. The obtained EV-containing pellet was then resuspended in 200 µL of PBS and either used immediately for EV characterization or stored at −80 °C until further use. For methodological comparison, an initial PPP volume of 900 µL was used.

#### 2.4.5. Exo-Spin^TM^ Exosome Blood Purification Kit

EVs were also isolated with the commercially available Exo-Spin^TM^ Blood Exosome Purification kit (Cell Guidance Systems, Cambridge, UK), according to manufacturer’s instructions. Briefly, 225 µL of plasma were centrifuged at 300 *g* for 10 min at 4 °C and the supernatant centrifuged at 16,000 *g* for 30 min at 4 °C. The supernatant was transferred into a new tube, mixed with the Buffer (from the commercial kit) in a 2:1 ratio and incubated for 1 h at 4 °C. Following incubation, samples were centrifuged at 16,000 *g* for 1 h at 4 °C, the supernatant was discarded, and the pellet was resuspended in 100 µL of PBS. Then, the columns (supplied by the kit) were equilibrated by adding 250 µL of PBS and centrifuging at 50 *g* for 10 s. Finally, the 100 µL of EV-resuspended pellet were added to the column and, following a centrifugation at the previously described conditions, eluted with 200 µL of PBS through a second centrifugation (50 *g*, 1 min, 4 °C). The recovered eluate was either used immediately for EV characterization or stored at −80 °C until further use. For methodological comparison, an initial PPP volume of 900 µL was used, which implied the need to use four columns (225 µL of PPP per column).

### 2.5. EV Characterization Methods

#### 2.5.1. Protein Quantification

Protein amounts were quantified using a Lowry-based protein detection method, according to the manufacturer’s instructions (DC™ Protein Assay kit; BioRad, Hercules, CA, USA; 5000116). BSA was used as a protein standard. After a minimum of 30 min incubation period in the dark, the absorbance was measured in a microplate reader (Synergy^TM^ Mx, Biotek Instruments Inc., Winooski, VT, USA) with a 488 nm excitation wavelength and a 655 nm emission wavelength. The data was obtained with the Gen5 Software and exported to the Microsoft Excel spreadsheet software for upcoming analysis.

#### 2.5.2. Nanoparticle Tracking Analysis (NTA)

Particle mean size and concentration were assessed by nanoparticle tracking analysis (NTA). According to manufacturer’s instructions, all samples were 1:1000–1:10,000 pre-diluted to reach the optimal particle concentration readout range (10^8^–10^9^ particles/mL). Samples were loaded into the instrument at a constant flow rate and at RT, using a 1 mL syringe (Omnifix^®^ 100 Solo; B|BRAUM, Melsungen, Germany) and a NanoSight syringe pump (Malvern Instruments Ltd.; Malvern, UK). Then, three separate 30 s videos were recorded, with the following specifications: Camera Type sCMOS; Laser Type Blue488; Camera Level 15; Slider Shutter 1206; Slider Gain 366; FPS 25.0; Temperature 20.4–24.0 °C; Viscosity 0.9 cP; Syringe Pump Speed 40. For methodological comparison, the following parameters were considered: Camera Type sCMOS; Laser Type Blue488; Camera Level 16; Slider Shutter 1300; Slider Gain 512; FPS 25.0; Temperature 22.6–25.5 °C; Viscosity 0.9 cP; and Syringe Pump Speed 40. Particles were detected by video analysis using a NanoSight NTA Software version (NTA 3.2 Dev Build 3.2.16) with the following settings: Detection Threshold 5; Blur Size Auto; and Max Jump Distance Auto (8.3–16.2 pixels).

#### 2.5.3. Transmission Electron Microscopy (TEM)

The size and morphology of the isolated EVs were assessed through negative staining transmission electron microscopy (TEM). EVs were pre-diluted with an HEPES 20 mM (Merck Life Science, Darmstadt, Germany; H4034) with a 4% (*w*/*v*) Sucrose (Merck Life Science, Darmstadt, Germany; S0389) solution (1:2) to allow proper visualization. Briefly, 10 μL of isolated EVs were mounted on Formvar/carbon film-coated mesh nickel grids (Electron Microscopy Sciences, Hatfield, PA, USA) for 2 min and dried with filter paper. Then, TEM grids were counter-stained with uranyl acetate for 10 s, after which liquid in excess was removed with filter paper. EVs were visualized using a JEM 1400 transmission electron microscope at 80 keV (JEOL, Tokyo, Japan). Images were digitally recorded using a Orious 1100W (Tokyo, Japan) CCD digital camera. Representative TEM photographs were acquired. This procedure, including EVs visualization, was carried out by the Histology and Electron Microscopy (HEMS) core facility, i3S, Porto, Portugal.

#### 2.5.4. Proteomic Analysis

For protein identification using proteomic analysis by liquid chromatography with tandem mass spectrometry (LC-MS/MS), 30 μg of protein from the isolated EVs and their respective cells of origin were prepared. Each sample was processed for proteomic analysis following the solid-phase-enhanced sample-preparation (SP3) protocol and enzymatically digested with Trypsin/LysC, as previously described elsewhere [45]. An equal amount of peptides (500 ng) was injected for each sample. Quantitative normalization was performed in Proteome Discoverer using the Total Peptide Amount method. Protein identification and quantitation was performed by nano-LC-MS/MS following an already published procedure [46]. Briefly, this equipment is composed by an Ultimate 3000 liquid chromatography system coupled to a Q-Exactive Hybrid Quadrupole-Orbitrap mass spectrometer (Thermo Scientific, Bremen, Germany). Samples were loaded onto a trapping cartridge (Acclaim PepMap C18 100 Å, 5 mm × 300 µm i.d., 160454, Thermo Scientific, Bremen, Germany) in a mobile phase of 2% ACN, 0.1% FA at 10 µL/minute. After a 3 min loading, the trap column was switched in-line to a 50 cm-by-75 μm inner diameter EASY-Spray column (ES803, PepMap RSLC, C18, 2 μm, Thermo Scientific, Bremen, Germany) at 250 nL/minute. Separation was generated by mixing A: 0.1% FA, and B: 80% ACN, with the following gradient: 5 min (2.5% B to 10% B), 120 min (10% B to 30% B), 20 min (30% B to 50% B), 5 min (50% B to 99% B) and 10 min (hold 99% B). Subsequently, the column was equilibrated with 2.5% B for 17 min. Then, for protein identification and quantification, the UniProt database was considered for the *Homo sapiens* (2021_03 with 20,371 entries), and *Bos taurus* (2021_03 with 6014 entries) reviewed proteomes together with a human spectral library (NIST_Human_Orbitrap_HCD_20160923). The acquired MS/MS data were processed using the Proteome Discoverer 2.5.0.400 software (Thermo Scientific, Bremen, Germany). Protein-label-free quantitation was performed with the Minora feature detector node at the processing step. Precursor ion quantification was performed at the processing step with the following parameters: (a) peptides—unique plus razor; (b) precursor abundance—based on intensity; (c) normalization mode—based on the total peptide amount; (d) pairwise protein ratio calculation; and (e) the hypothesis test—based on a *t*-test (background based). The LC-MS/MS analysis, as well as, raw-data processing were carried out by the Proteomics scientific platform, i3S, Porto, Portugal. The contents of OCI-AML3 cells and their respective isolated EVs were analyzed in three independent experiments. In the end, the results (average of the experiments performed) were analyzed considering several parameters: (1) The proteins whose identified organism was *Bos taurus* were not considered. (2) Common contaminants (MaxQuant Common Repository of Adventitious Proteins database) were excluded; (3) a filter was applied for “unique peptides” greater or equal to 2; and (4) false-discovery rate (FDR) was set to “high” confidence. The presence of LAPMs in the EV cargo was compared with their presence in the corresponding cells of origin. Only proteins identified with a confidence level of at least “High” in Proteome Discoverer and consistently detected across all three EV biological replicates were included in the analysis. The mass spectrometry proteomics data have been deposited to the ProteomeXchange Consortium via the PRIDE partner repository with the dataset identifier PXD071884 [47].

#### 2.5.5. Gene Ontology by PANTHER Analysis

The Gene Ontology analysis of the proteins identified by proteomics was elucidated with the PANTHER Classification System (version 17.0 released 22 February 2022) [48]. UniProtKB entries were used as the unique identifiers of the proteins and were submitted into this program, and all proteins were compared to a reference proteome dataset (*Homo sapiens*) provided by PANTHER to identify their biological processes and molecular functions.

#### 2.5.6. Western Blot (WB)

For protein detection by Western blot (WB), all samples were first prepared by adding loading buffer (Tris-HCl 1 M pH 6.8, 10% SDS, 85% glycerol, β-mercaptoethanol, 1% bromophenol blue) and boiling at 95 °C for 5 min. An equal amount of protein for each experimental condition (15 µg) was loaded and separated in a sodium dodecyl sulfate polyacrylamide gel at a % that depended on the proteins analyzed (10% acrylamide gel for separating 20 kDa to 300 kDa proteins and 12% acrylamide gel for separating 10 kDa to 200 kDa proteins) for 30 min at 70 V, and for approximately 1 h at 100 V, and transferred to a nitrocellulose membrane (GE Healthcare Life science, Chalfont St Giles, UK; GE10600002) for 90 min at 100 V. To verify successful protein transfer, membranes were initially stained with Ponceau S solution (PanReac AppliChem, Barcelona, Spain; A2935). They were then incubated for 2 h at room temperature on a Standard Analog Shaker (VWR, Lutterworth, UK) in Tris-buffered saline (TBS, pH 7.4; 80 g NaCl, 2 g KCl, and 30 g Tris-Base, Merck Life Science, Darmstadt, Germany; T6066) containing 0.1% Tween^®^ 20 (Promega, H5152) and 5% (*w*/*v*) non-fat dry milk (Molico, Nestlé, Vevey, Switzerland) to block nonspecific binding, as described elsewhere [49]. Membranes were then incubated with agitation overnight at 4 °C or for 90 min at RT with the following primary antibodies: anti-Albumin (1:200; Santa Cruz Biotechnology, Dallas, TX, USA; sc-271605), anti-ApoB (1:200; Santa Cruz Biotechnology, Dallas, TX, USA; sc-13538), anti-Actinin-4 (1:200; GeneTex, Irvine, CA, USA; GTX113116), anti-Alix (1:200; Santa Cruz Biotechnology, Dallas, TX, USA; sc-53540), anti-HSP70 (1:200; Santa Cruz Biotechnology, Dallas, TX, USA; sc-66048), anti-TSG101 (1:200, Santa Cruz Biotechnology, Dallas, TX, USA; sc-136111), anti-Syntenin-1 (1:100; Santa Cruz Biotechnology, Dallas, TX, USA; sc-100336, sc-515538), anti-CD63 (1:200; Santa Cruz Biotechnology, Dallas, TX, USA; sc-5275; 1:1000; SBI Biotech, Tokyo, Japan; EXOAB-CD63A-1), anti-CD81 (1:200; Santa Cruz Biotechnology, Dallas, TX, USA; sc-7637; 1:1000; SBI Biotech, Tokyo, Japan; EXOAB-CD81A-1), anti-Integrin αM (1:100; Santa Cruz Biotechnology, Dallas, TX, USA; sc-515923), anti-CD13 (1:100; Santa Cruz Biotechnology, Dallas, TX, USA; sc-166105), anti-CD14 (1:200; Santa Cruz Biotechnology, Dallas, TX, USA; sc-58951), anti-CD33 (1:1000; abcam, Cambridge, UK; ab134115), anti-CD34 (1:10,000; abcam, Cambridge, UK; ab81289), anti-CD35 (1:200; Santa Cruz Biotechnology, Dallas, TX, USA; sc-7308), anti-c-Kit (1:200; Santa Cruz Biotechnology, Dallas, TX, USA; sc-365504), anti-IL-3Rα (1:200; Santa Cruz Biotechnology, Dallas, TX, USA; sc-455), anti-HLA-DR (1:200; Santa Cruz Biotechnology, Dallas, TX, USA; sc-53319), anti-MPO (1:200; Santa Cruz Biotechnology, Dallas, TX, USA; sc-52707), anti-B23 (1:200; Santa Cruz Biotechnology, Dallas, TX, USA; sc-47725), and anti-TdT (1:100; Santa Cruz Biotechnology, Dallas, TX, USA; sc-393710). After washing steps with TBS-T, membranes were incubated with the secondary antibodies: mouse anti-goat IgG-HRP (1:2000; Santa Cruz Biotechnology, Dallas, TX, USA; sc-2354), anti-rabbit IgG-HRP (from donkey) (1:2000; GE Life Sciences, Cytiva, Marlborough, MA, USA; NA934), goat anti-rabbit IgG-HRP (1:20,000; SBI Biotech, Tokyo, Japan; EXOAB-HRP) or anti-mouse IgG-HRP (from sheep) (1:2000; GE Life Sciences, Cytiva, Marlborough, MA, USA; NA931) for 1 h at RT, in an orbital shaker. Following washes with TBS-T, peroxidase activity was visualized using Amersham Hyperfilm ECL (Cytiva, Marlborough, MA, USA; 28906835). Chemiluminescent signals were developed with a Fuji Medical Film Processor (Model FPM-100A; Fuji Photo, Tokyo, Japan). Immunoblot images were subsequently digitized using a GS-800 Calibrated Densitometer (Bio-Rad, Hercules, CA, USA; 170-7980).

### 2.6. Statistical Analysis

Each experiment was independently repeated at least three times. Results are reported as the mean value with the corresponding standard deviation. Statistical comparisons were carried out using an unpaired, two-sided t-test in GraphPad Prism (version 8.0). A probability value below 0.05 was taken as evidence of statistical significance.

## 3. Results

### 3.1. Isolation and Characterization of EVs Released by OCI-AML3 AML Cells

Our work aimed to explore the possibility of using AML-derived EVs as a source of MRD biomarkers. Having this in mind, we started by verifying whether EVs released by a human AML cell line (OCI-AML3) carry LAPMs in their cargo. For that, following a 72 h incubation period in medium supplemented with EV-depleted serum, EVs were isolated from the conditioned medium of OCI-AML3 cells following a modified differential centrifugation protocol [43]. In order to confirm their successful isolation, the isolated EVs were further characterized according to their morphology, by transmission electron microscopy (TEM), and size and concentration, by nanoparticle tracking analysis (NTA).

**Figure 1:**
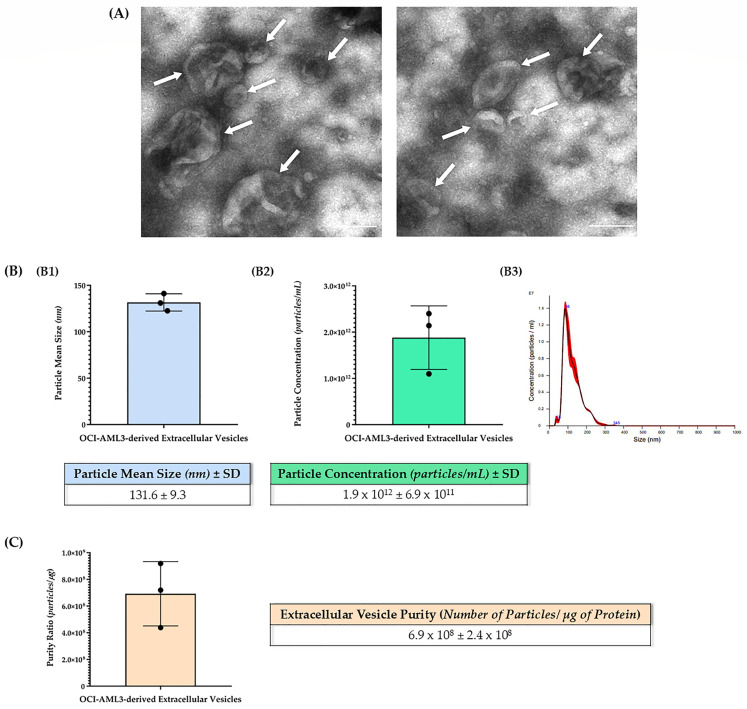
Characterization of EVs isolated by differential centrifugation from AML OCI-AML3 cells conditioned medium. (**A**) Morphology of EVs (arrowheads) isolated from OCI-AML3 cells, analyzed by transmission electron microscopy (TEM). Bar = 200 nm. (**B**) Particle mean size (**B1**) and concentration (**B2**) measured by nanoparticle tracking analysis (NTA). Results are representative of the mean ± SD of at least three independent experiments. (**B3**) Particle concentration (left axis) plotted against particle mean size (right axis), both measured by NTA. Representative graphical representation for one independent experiment. (**C**) Purity ratio of the EV preparations obtained by differential centrifugation. Purity ratio is obtained by the ratio between particle concentration (measured by NTA) and protein concentration (assessed by a modified Lowry method). Results are representative of the mean ± SD of at least three independent experiments.

Results from TEM (Figure 1A) revealed that the OCI-AML3 cells released particles which were within the typical size range of EVs (nanoscale) and that the observed particles presented a typical spherical and cup-shaped morphology, confirming that there was no degradation of the isolated EVs. Of note, the observed cup-shaped appearance has been reported to be a technical artefact derived from the fixation process, as a result of dehydration [50]. The obtained TEM images, however, also demonstrated the presence of some protein contaminants (black spots in Figure 1A). Regarding the NTA results (Figure 1B), the particle mean size detected for OCI-AML3-derived particles was 131.6 nm, thus being within the typical size range of EVs. Furthermore, a concentration of 1.9 × 10^12^ particles/mL was detected for the obtained EV preparation. The purity of the obtained EV preparations (Figure 1C) was calculated through the ratio between particle concentration (assessed through NTA) and protein concentration (measured by a modified Lowry method), as previously described [51].

### 3.2. Protein Content Analysis of AML Cell-Derived EVs and of Their Corresponding Releasing Cells

#### 3.2.1. Detection of Well-Established EV-Related Protein Markers

In order to confirm that the particles isolated from the OCI-AML3 conditioned medium were EVs, the presence of well-established EV-associated protein markers (described by the MISEV2023 guidelines [28]) was evaluated by quantitative proteomic analysis and further confirmed by WB. According to the MISEV2023 guidelines, in order to prove the presence of EVs, it is recommended to analyze at least one marker corresponding to a (1) transmembrane protein associated to the plasma membrane (e.g., CD9, CD63, CD81) to confirm the presence of the lipid-bilayer structural characteristic of EVs and (2) cytosolic protein with membrane-binding-capacity, to show that that lipidic structure is incorporating intracellular material (e.g., TSG101, ALIX, syntenin) [28]. Furthermore, in studies that focus on one or more EV subtypes, it is recommended to analyze at least one marker corresponding to a (3) transmembrane, lipid-bound and soluble protein associated to other intracellular compartments than plasma membrane/endosomes (e.g., Actinin-1/4), which are representative of large EVs subtype [28,52].

Here, several established EV-associated protein markers indicated by the MISEV2023 guidelines [28] were detected in EVs released by the OCI-AML3 cell line, and the identified proteins categorized as “high confidence” or “peak found”. Indeed, as shown in Table 1, among the most abundant proteins identified in the isolated pellet, a total of 15 proteins that demonstrate the presence of EVs were detected. For instance, basigin (BSG), integrin beta-1 (ITGB1), CD81 and integrin alpha-5 (ITGBA5), which are included in the previously mentioned category (1), and heat shock protein HSP 90-beta (HSP90AB1), syntenin-1 (SDCBP), programmed cell death 6-interacting protein (PDCD6IP, also known as ALIX), and glyceraldehyde-3-phosphate dehydrogenase (GAPDH), which are included in the category (2), were detected in the analyzed EV preparation. Furthermore, α-actinin 4, which is included in the previously mentioned category (3), was also detected in the isolated pellet.

**Figure 2:**
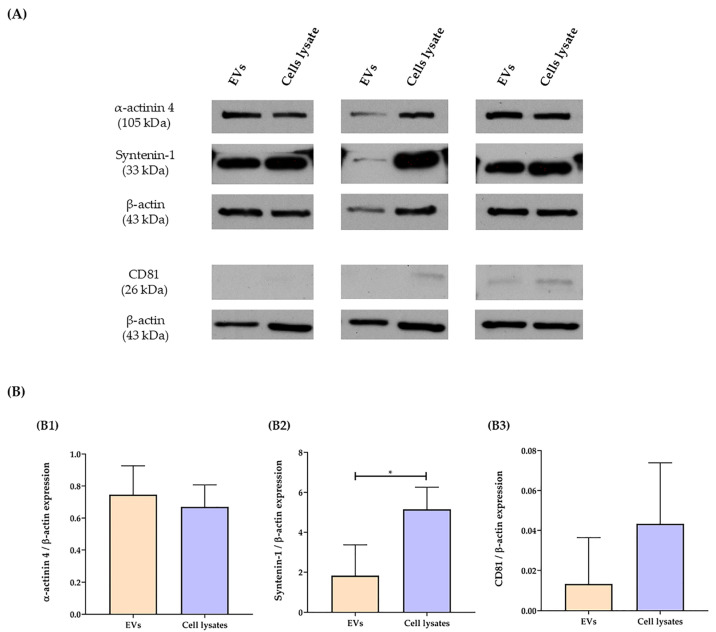
Validation by Western blot analysis of the presence of some EV-associated protein markers initially identified by quantitative proteomic analysis. (**A**) Presence of the EV-associated protein markers α-actinin 4, syntenin-1 and CD81 in EVs isolated from the OCI-AML3 cell line and in their cells of origin. Blots are representative of at least 3 independent experiments. β-actin was used as loading control. Ponceau staining was also used to confirm the gels loading (data not shown). (**B**) Protein levels normalized against β-actin: α-actinin 4 (**B1**), syntenin-1 (**B2**) and CD81 (**B3**). Results are representative of the mean ± SD of at least three independent experiments. * *p* < 0.05.

The presence of some established EV protein markers identified by this proteomic analysis (Table 1) was confirmed by WB analysis (Figure 2). Results demonstrated that the pellet isolated by differential centrifugation was positive for α-actinin 4, CD81 and syntenin-1, confirming the EV nature of the pellet content. The β-actin was used as a loading control for cells but was also detected in the EVs, being present in all samples. Ponceau staining (data not shown) was used to confirm the gels loading.

In particular, the presence of α-actinin 4 was confirmed in all three replicates, and, according to Figure 2(B1), which represents the normalized expression levels against β-actin expression, α-actinin 4 expression levels were similar in EVs and in their releasing cells. The presence of syntenin-1 was also confirmed in all samples, and, as shown in Figure 2(B2), syntenin-1 levels were significantly higher in cell lysates than in their corresponding EVs, which was not expected. Accordingly, the presence of CD81 was also confirmed in EVs, and the analysis of its normalized expression levels (Figure 2(B3)) demonstrated that CD81 levels were also higher (although not significantly) in the OCI-AML3 cells than in their released EVs.

Therefore, through four different approaches (TEM, NTA, quantitative proteomic analysis and WB analysis) it was proven that the pellet isolated by differential centrifugation contained EVs. Thus, further downstream analyses of protein cargo were carried out in those EVs.

#### 3.2.2. Detection of Leukemia-Associated Phenotypic Protein Markers (LAPMs)

As previously mentioned, one of the main goals of this work was to determine whether EVs released by AML cell lines carried LAPMs in their cargo. As shown in Table 2, a total of three clinically described LAPMs were found by proteomic analysis of the OCI-AML3 cells. In fact, CD2BP2 was detected with “high confidence” in all three replicates of the OCI-AML3 cells; CD14 was detected with “high confidence” in two replicates and as a “peak found” in one replicate; and CD33 was detected with “high confidence” in one replicate, as a “peak found” in another replicate and was “not found” in the third replicate of cells. Importantly, two out of these three LAPMs were also detected in the cargo of OCI-AML3-released EVs (Table 2). Indeed, both CD14 and CD33 were detected with “high confidence” in one replicate of EVs and as a “peak found” in the other two replicates.

**Figure 3:**
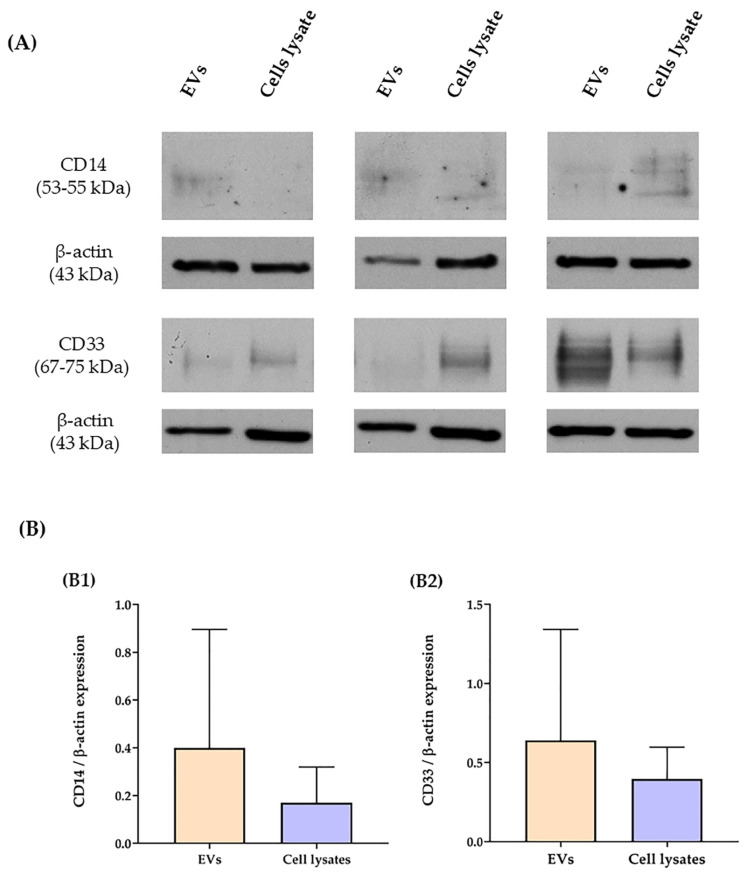
Validation by Western blot analysis of the presence of LAPMs initially identified by quantitative proteomic analysis. (**A**) Presence of LAPMs CD14 and CD33 isolated from the OCI-AML3 cell line. Blots are representative of at least 3 independent experiments. β-actin was used as loading control. Ponceau staining was also used to confirm the gels loading (data not shown). (**B**) Protein levels were normalized against β-actin: CD14 (**B1**) and CD33 (**B2**). Results are representative of the mean ± SD of at least three independent experiments.

Next, we proceeded with a WB analysis to confirm whether the LAPMs identified by quantitative proteomic analysis of the OCI-AML3 cells and their released EVs were indeed present in those samples (Figure 3). CD14 and CD33 were present (even though with high variability) in all three replicates of both OCI-AML3 cells and their shed EVs.

#### 3.2.3. Proteins Enriched in the EVs Released by the OCI-AML3 Cells

**Figure 4:**
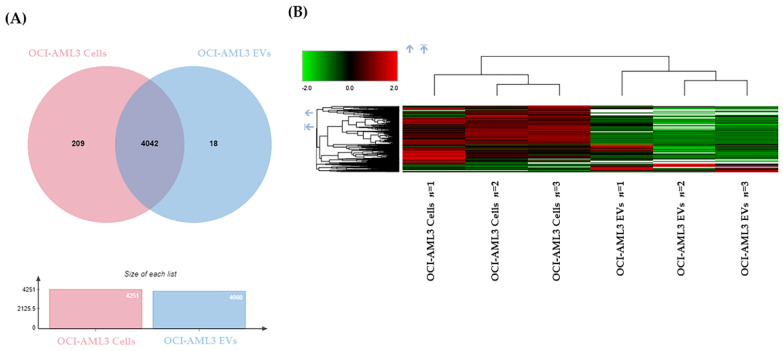
Quantitative proteomic analysis of OCI-AML3 cells and their released EVs. (**A**) Venn diagram showing the number of “overlapping” and “unique” proteins detected on the OCI-AML3 cells and their shed EVs. The Venn diagram was obtained through the Jvenn program [53]. (**B**) Heat Map of the protein content of the OCI-AML3 cells *versus* the protein content of OCI-AML3-derived EVs, obtained through the Proteome Discoverer 2.5.0.400 software (Thermo Scientific).

As depicted in Figure 4A, 4042 proteins were identified in both OCI-AML3 cells and their released EVs (either with “high confidence” or “peak found”), with 209 proteins only found present in the cells and 18 proteins exclusively found in EVs. This means that, as expected, most of the proteins found in EVs derived from the cells of origin. Furthermore, the Heat Map analysis (Figure 4B), in which the red color highlights enriched protein families and the green color represents the low presence of protein families, demonstrated the similarity of the protein content between the three replicates (of cells or EVs). The Heat Map also highlights the differences in the protein content between cells and EVs.

#### 3.2.4. Gene Ontology Analysis of EV Protein Cargo

**Figure 5:**
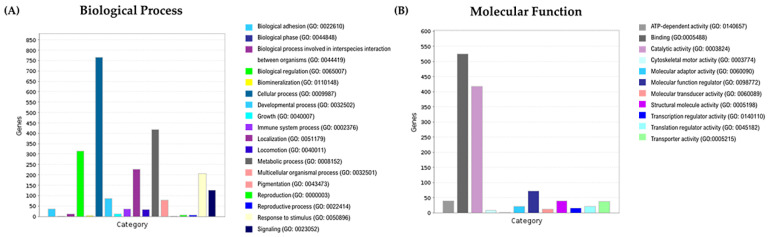
Gene Ontology analysis of the proteins found present with high confidence in EVs released by the OCI-AML3 cell line, regarding the biological process (**A**) and molecular function (**B**) ontologies. Bar graphs were obtained through the PANTHER classification system [48].

Regarding the number of proteins present in the EVs shed by the OCI-AML3 cell line, with high confidence and present in all three replicates, a total of 1243 proteins were detected. As shown in Figure 5, Gene Ontology analysis revealed that those proteins are mainly involved in cellular or metabolic processes (Figure 5A), and the most significantly enriched molecular functions are related with binding or catalytic activity (Figure 5B).

As for the biological processes, cellular metabolic process (cellular processes) and organic substance metabolic process (metabolic processes) are the most significantly enriched categories (Supplementary Figure S1). Regarding the molecular functions, the categories most abundant are protein binding (binding functions) and hydrolase activity (catalytic activity functions) (Supplementary Figure S2).

Additionally, the most abundant proteins present in EVs released by the OCI-AML3 cell line were also analyzed (Supplementary Table S1). Corroborating our Gene Ontology analysis, the most abundant proteins present in EVs are (i) Moesin (MSN), Non-POU domain-containing octamer-binding protein (NONO), Heat shock protein HSP 90-beta (HSP90AB1), alpha-enolase (ENO1), alpha-2-HS-glycoprotein (AHSG), and [F-actin]-monooxygenase (MICAL1), which are associated with cellular processes; (ii) NONO, ENO1, and AHSG, which are associated with metabolic processes; (iii) MSN, HSP90AB1, AHSG, and MICAL1, which have binding functions; and (iv) ENO1 and AHSG, which have catalytic activity functions.

### 3.3. Selection of an Appropriate Method for EV Isolation from the PB of AML Patients

Once it was established that LAPMs were present in the cargo of EVs shed by leukemic cells *in vitro*, we proceeded to search for an appropriate methodology for EV isolation from the PB of AML patients. With that in mind, the profiles of EVs derived from AML patients’ poor platelet plasma (PPP) were evaluated following isolation through three distinct methodologies: one column-based method, Size Exclusion Chromatography followed by Ultrafiltration (SEC-UF), one column- and precipitation-based method, Exo-Spin^TM^ Exosome Blood Purification Kit (Cell Guidance Systems), and one precipitation-based method, Total Exosome Isolation Kit (Invitrogen). For comparison purposes, an equal initial PPP volume was used (900 µL). Three patients’ samples collected at diagnosis were analyzed with each methodology under study. Then, similarly to what was previously described for the EVs isolated from the cell line, the patients’ isolated EVs were characterized according to their size, morphology and protein cargo.

Regarding the EV isolation recovery volume and time required to complete the protocol (including incubation periods), the commercially available Exo-Spin^TM^ Exosome Blood Purification Kit provided a bigger final volume of EV preparation (roughly 800 µL), whereas the commercial precipitation-based method Total Exosome Isolation Kit provided a faster EV isolation (Table 3).

**Figure 6:**
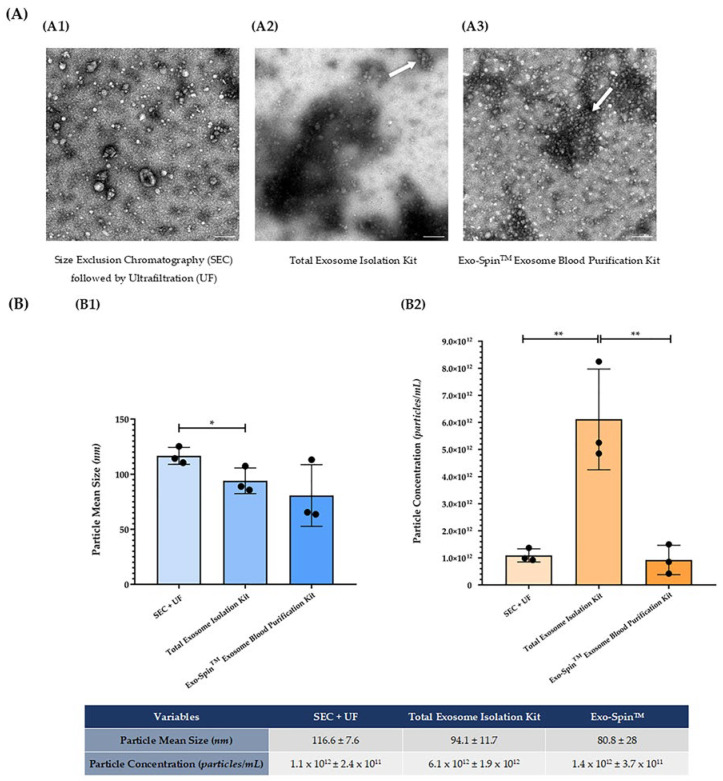
Characterization of EVs isolated from patients’ PPP. (**A**) Morphology of EVs isolated from PPP by (**A1**) size exclusion chromatography followed by ultrafiltration (SEC-UF), (**A2**) Total Exosome Isolation Kit and (**A3**) Exo-Spin^TM^ Exosome Blood Purification Kit, analyzed by transmission electron microscopy (TEM). Bar = 200 nm. (**B**) Particle mean size (**B1**) and concentration (**B2**) measured by nanoparticle tracking analysis (NTA). Results are representative of the mean ± SD of at least three independent experiments. * *p* < 0.05, ** *p* < 0.01.

Results from TEM demonstrated that all three methodologies successfully isolated EVs within the expected size (nanoscale) and morphology (Figure 6A). Nevertheless, images obtained from EVs isolated using the commercially available Total Exosome Isolation Kit and Exo-Spin^TM^ Exosome Blood Purification Kit pointed out the presence of a greater amount of protein contaminants (black spots in Figure 6(A2,A3)) and EV aggregates (arrowheads in Figure 6(A2,A3)) in these preparations.

With regard to the NTA results, particle mean sizes of 116.6 ± 7.6 nm, 94.1 ± 11.7 nm and 80.8 ± 28 nm were detected for the SEC-UF, the Total Exosome Isolation Kit and the Exo-Spin^TM^ Exosome Blood Purification Kit, respectively (Figure 6(B1)). Thus, although a slight reduction in the particle mean size of EVs isolated through the commercially available kits when compared to the EVs isolated by SEC-UF has been observed, for all three methodologies the particle mean size was similar and within the typical size range of EVs. Interestingly, as demonstrated in Figure 6(B2)**,** particle concentration (particles/mL) was significantly higher (*p* < 0.01) when using the commercial Total Exosome Isolation Kit (6.1 × 10^12^ ± 1.9 × 10^12^ particles/mL).

**Figure 7:**
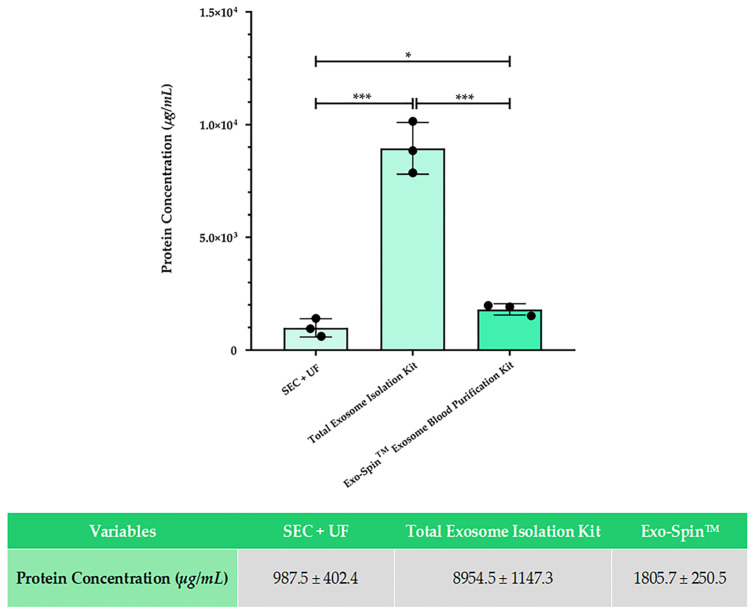
Protein concentration (µg/mL) of particles isolated with 3 methods (SEC-UF, Total Exosome Isolation Kit and Exo-Spin^TM^ Exosome Blood Purification Kit) measured by a modified Lowry method. Results are representative of the mean ± SD of at least three independent experiments. * *p* < 0.05, *** *p* < 0.001.

Accordingly, results from protein quantification (modified Lowry method) detected a considerably higher protein concentration (µg/mL) in the EV preparations obtained using the Total Exosome Isolation Kit, when compared to the amount of protein detected with the other methodologies under study (Figure 7). Nevertheless, the observed elevated levels of protein could be related to the presence of a greater amount of protein contaminants in these EV preparations, rather than to elevated levels of protein EV-cargo, as suggested by the previously presented TEM results.

**Figure 8:**
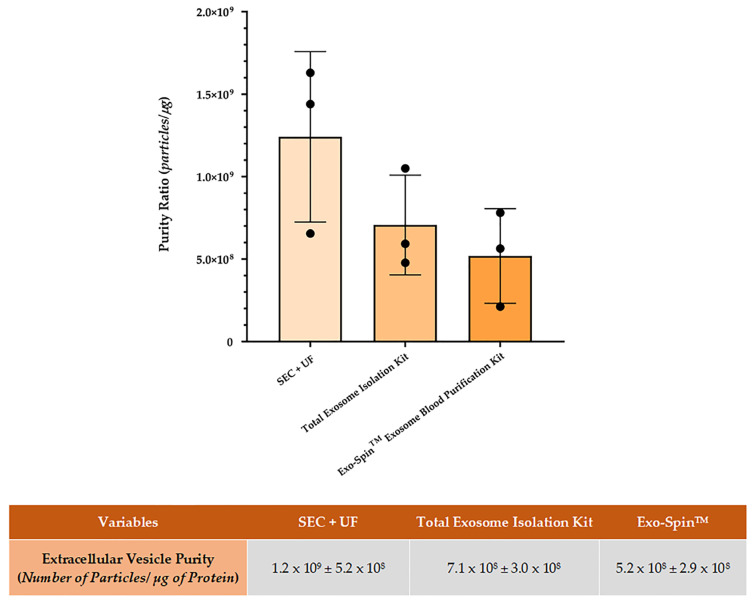
Purity ratio of the EV preparations obtained by size exclusion chromatography followed by ultrafiltration (SEC-UF), Total Exosome Isolation Kit and Exo-Spin^TM^ Exosome Blood Purification Kit. Purity ratio is obtained by the ratio between particle concentration (measured by NTA) and protein concentration (assessed by a modified Lowry method). Results are representative of the mean ± SD of at least three independent experiments.

Indeed, the purity of the obtained EV preparations was calculated through the ratio between particle concentration (assessed through NTA) and protein concentration (measured by a modified Lowry method), as previously described [51]. Results (Figure 8) demonstrated that EV preparations obtained by SEC-UF were the ones presenting higher purity ratios, whereas EV preparations isolated by the Exo-Spin^TM^ Exosome Blood Purification Kit were the ones exhibiting lower purity.

**Figure 9:**
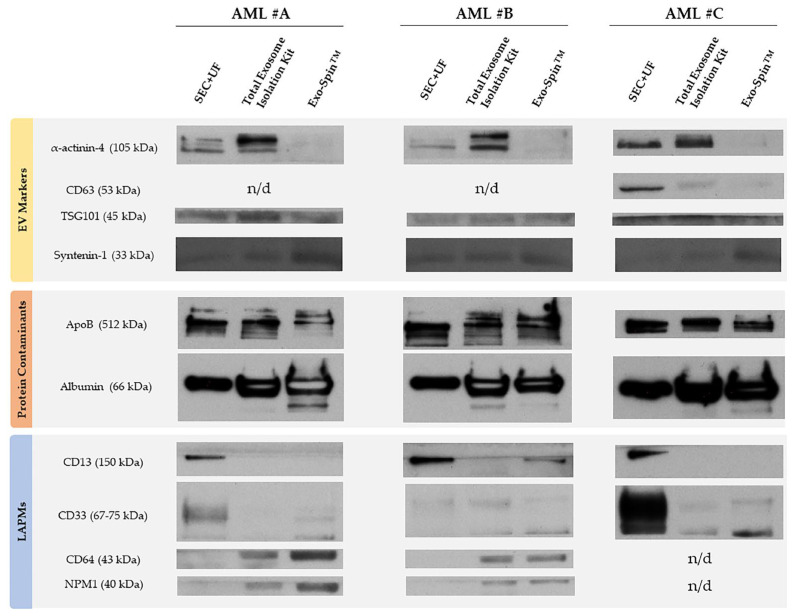
Western blot analysis of the presence of EV-associated protein markers (α-actinin 4, CD63, TSG101 and syntenin-1), protein contaminants (ApoB and albumin) and some LAPMs (CD13, CD33, CD64 and NPM1) in EVs, isolated by size exclusion chromatography followed by ultrafiltration (SEC-UF), Total Exosome Isolation Kit, or Exo-Spin^TM^ Exosome Blood Purification Kit from AML patients’ PPP, is displayed. The same amount of EV protein was loaded in all conditions. Ponceau staining was used to confirm the gels loading (data not shown). Representative results obtained from three AML patients: AML#A, #B and #C. n/d: non-detected.

Taking into consideration the previously presented results, we proceeded to perform a WB analysis to verify the levels of some well-established EV markers (α-actinin 4, CD63, TSG101 and syntenin-1) as well as plasma protein contaminants (ApoB and Albumin). Results, presented in Figure 9, confirmed the EV nature of the EV preparations obtained by all three methodologies. In agreement with the results obtained regarding EV size (Figure 6(B1)), the detected levels of α-actinin 4, a well-established marker for the large EVs subtype, were considerably lower in the EV preparations obtained by the Exo-Spin^TM^ Exosome Blood Purification Kit, suggesting that this sub-population of EVs is not present (or is present in small amounts) in these EV preparations. This is explained by the fact that this is an exosome isolation method (supposedly only isolating a population of EVs ranging in size from 30 to 150 nm) [27].

As expected, a higher expression of ApoB and albumin (plasma protein contaminants) was detected for EV preparations obtained using the Total Exosome Isolation Kit and the Exo-Spin^TM^ Exosome Blood Purification Kit.

Interestingly, when the levels of some LAPMs were evaluated, results showed that their detection depends on the methodology used and that certain LAPMs are detected with one methodology but not with the other(s). This raises a major issue when analyzing LAPMs in EVs isolated from blood plasma, leading us to the conclusion that the same methodology should be used for longitudinal comparative analysis of patients.

Given the demonstrated superior purity of EVs isolated by size exclusion chromatography followed by ultrafiltration (SEC-UF), further analysis was performed through this column-based method only.

### 3.4. Presence of LAPMs in AML Patient’s Plasmatic EVs

As previously referred, with this work we intended to study the potential applicability of a PB EV-based liquid biopsy to detect MRD biomarkers in AML patients. To this end, we next proceeded to evaluate whether clinically established LAPMs were present in the AML patients’ plasmatic EVs and to explore whether these AML markers levels vary throughout different evolutionary states of the disease. This study did not intend to compare the advantages of this approach with the currently used approach to detect MRD in AML patients; instead, the current work intended to carry out a proof-of-concept study to verify if such a comparison study would be feasible in the future.

Therefore, EVs were isolated from seven AML patients’ blood plasma collected at diagnosis and after induction chemotherapy, when a morphological complete remission was produced; three patients also had samples collected at relapse. Their content in a panel of clinically established LAPMs (CD35, CD13, CD11b, CD117, CD34, MPO, CD123, CD33, Tdt, CD14, NPM1 and HLA-DR) was then evaluated by WB analysis. Cell lines lysates (from KG1a and HL60) were used as positive controls in all WB analysis to confirm molecular weights of proteins/bands (data not shown).

**Figure 10:**
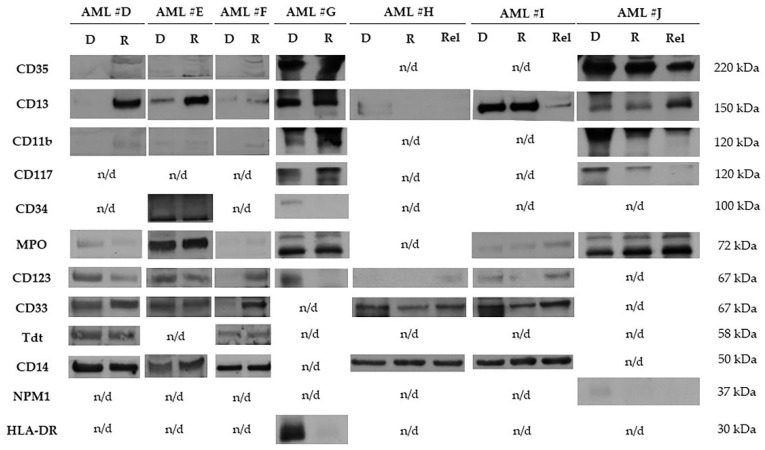
Western blot analysis of the presence of LAPMs in EVs isolated from seven AML patient’s (AML #D to #J) blood plasma. Analysis of CD35, CD13, CD11b, CD117, CD34, MPO, CD123, CD33, Tdt, CD14, NPM1 and HLA-DR was performed in EVs isolated from AML PB samples collected at diagnosis and complete remission (and at relapse for three patients). The collected SEC fractions F3 to F6 were pooled together, ultrafiltrated and quantified. The same amount of EV protein (15–25 µg, depending on the patient) was loaded onto the gel, when comparing paired diagnostic/remission samples. Ponceau staining was used to confirm the gels loading (data not shown). n/d: non-detected.

Results (Figure 10) demonstrated that the analyzed LAPMs were detected in the majority of patient plasma-derived EVs. For instance, as expected, the myeloid lineage marker CD13 was detected in EVs isolated from the PB of all patients included in this study. Furthermore, analysis of the same protein marker at distinct stages of the disease (diagnosis, complete remission and, in some patients, relapse) revealed a variable level of each analyzed marker over the clinical evolution of the disease.

For patients #D, #E, #F and #G, relapse samples were not available for analysis. Therefore, for these patients the presence of LAPMs in circulating plasmatic EVs was only analyzed at diagnosis and complete remission stages. As expected, some LAPMs decreased their detection levels following induction chemotherapy. Indeed, in patient #D both MPO and CD123 detection levels were slightly decreased at complete remission when compared to diagnosis. Furthermore, in patient #G the circulating EVs’ CD34, CD123 and HLA-DR levels decreased at complete remission when compared to their corresponding diagnosis sample. Curiously, some patients presented at least one marker with increased expression at complete remission. For instance, in patients #D and #E CD13 was increased at complete remission when compared to diagnosis. In patient #F the plasmatic EVs’ CD123 and CD33 levels were also increased at complete remission.

Notably, in patients for whom relapse samples were available for analysis (#H, #I and #J) some of the markers had their levels decreased at the remission stage: CD13 and CD33 in patient #H, CD123 and CD33 in patient #I, and CD35, CD117 and NPM1 in patient #J. Curiously, patient #I revealed a clear decrease at relapse of a marker initially slightly augmented (CD13) following induction chemotherapy. Most interestingly, some markers with decreased levels at remission were increased at relapse: CD33 in patient #H; CD123 and CD33 in patient #I; and CD13 in patient #J. The elevated levels of CD123 found at relapse in patient #I could be related to a potential immunophenotypic shift of the leukemic clone responsible for the relapse. Our approach can be patient specific, allowing the detection of markers that are only present in some of the AML patients or even at some point of the clinical evolution. To confirm this hypothesis, an extensive correlation between these results and the clinical multi-parameter flow cytometry results obtained for each patient, at each state of the disease, must be performed in future work.

## 4. Discussion

AML persists as the most commonly diagnosed acute leukemia among adults, with a total of 20.050 new cases estimated to occur in the United States in 2022 [1,54]. Despite the attempts to improve our understanding of this highly heterogeneous and complex disease, no major improvements in adults disease-free and overall survival have been made, and it remains associated with a poor 5-year survival rate [55]. This is related to the persistence of morphologically undetectable residual amounts of leukemic cells upon remission—MRD—which translates into a high frequency of post-treatment relapse [8,9]. Therefore, MRD monitoring has been pointed out as a valuable post-treatment prognostic indicator that must be carefully addressed. Nevertheless, current MRD monitoring requires the use of invasive BM aspirates, with a detrimental impact on regular and continuous disease monitoring [18,19]. Hence, the potential of minimally invasive liquid biopsies has been suggested, as they would allow real-time monitoring of the disease [21]. However, the use of PB with current methodologies is generally not recommended because of the lower detectable frequency of MRD [9]. Indeed, the clinical utility of circulating tumor cells (CTCs) is limited by their extreme scarcity in the bloodstream, where they are vastly diluted among normal hematologic cells (approximately 1 per 10^6^–10^9^) [56]. This rarity muddles reliable enrichment, characterization, and quantification, leading to technical variability and reduced robustness [56].

In contrast, EVs are abundant in a wide range of body fluids and may more accurately reflect intratumoral heterogeneity, with the potential to increase sensitivity in MRD monitoring [56]. As a matter of fact, over the past few years, EVs have been pointed out as potential sources of disease biomarkers in liquid biopsies [26]. In addition to being available in a multitude of biological fluids, including PB, the role of these nanosized particles in several physiological and pathological processes has been extensively described, and evidence demonstrated that EVs carry various cell-derived bioactive molecules in their cargo that can reflect characteristics of their cells of origin [25,26,29]. Therefore, MRD monitoring through a PB EV-based liquid biopsy approach could be of great interest to provide early insights into treatment efficacy as well as to allow patient risk stratification and relapse prediction.

In this work, we provided proof-of-concept evidence that AML-derived EVs carry proteins that could have the potential to be applied for longitudinal real-time disease monitoring.

Using quantitative proteomic analysis, we were able to demonstrate the presence of two LAPMs, CD14 and CD33, in the cargo of EVs isolated from the OCI-AML-3 cell line, which were also present in their leukemic cells of origin. Oddly, 18 proteins were exclusively found in the cargo of EVs but not in their cells of origin, which may be explained by two hypothesis: (1) all those proteins were selectively packaged into the EVs by their cells of origin, with none remaining in the cells, or (2) those proteins were not found in cells due to technical limitations of the proteomic analysis. This could be confirmed by performing detection of those proteins by other approaches, but this is outside the scope of the present work.

Recently, a strong correlation between the levels of AML MRD found in BM and PB samples was described, suggesting that measuring MRD in PB rather than in BM aspirates may be a suitable strategy for MRD monitoring [22]. Here, we proceeded to isolate and characterize EVs from the PB of AML patients, through three distinct isolation methodologies: size-exclusion chromatography followed by ultrafiltration (SEC-UF), Total Exosome Isolation Kit and Exo-Spin^TM^ Exosome Blood Purification Kit. Of note, we isolated EVs with these procedures and characterized those EVs according to their size, quantity and EV-protein cargo, but it is not possible to know what types of EVs (according to their biogenesis) were isolated with these different procedures. Through the comparison of the profiles of plasmatic circulating EVs isolated by each of the above mentioned techniques, it was demonstrated that SEC-UF was the one providing EV preparations with a smaller amount of EV aggregates and with highest purity ratios (particles/µg). These results are in agreement with those described in the literature, as SEC has been described to provide EVs with higher purity, less aggregation, and undamaged structural integrity [57]. WB analysis for the presence of LAPMs in plasmatic EVs revealed that all three methodologies under study allowed the detection of LAPMs in EVs isolated from the PB of AML patients at diagnosis. However, it was observed that depending on the methodology used, certain LAPMs may or may not be detected, suggesting that the same methodology should be used for comparative longitudinal analysis. In this study, we decided to proceed with SEC-UF for our subsequent analysis, due to the higher purity of the EVs obtained with this protocol. However, we acknowledge that the possibility of plasma protein contamination contributing to LAPM false-positive detection cannot be excluded for any of the methodologies under study.

In order to study the possible application of a PB EV-based liquid biopsy to detect MRD biomarkers in AML patients, we proceeded to analyze the presence of clinically established LAPMs in EVs isolated from AML patients’ PB samples collected at diagnosis, complete remission and relapse. Our results demonstrated that the analyzed LAPMs were detected in the majority of patient plasma-derived EVs and, importantly, that the majority of the analyzed LAPMs varied their expression through disease evolution. To our knowledge, this possibility of detecting different levels of LAPMs in the cargo of plasma EVs throughout disease evolution is innovative. However, contrary to what would be expected, most patients had at least one LAPM increased at complete remission. Although unexpected, this could be explained by four hypothesis. First, given the reported lack of consensus regarding the time that EVs may remain in circulation, it is possible that the isolated leukemia-derived EVs may not be representative of the disease burden at the time of collection [29,58]. Second, the observed increases in the levels of LAPMs could be related to hematopoietic recovery following induction chemotherapy [59]. Third, this may be an indicator of MRD. In fact, it is possible that the immunophenotypic evaluation of the bone marrow used in the clinic does not represent the tumor heterogeneity, and therefore an immunophenotypic evaluation of circulating EVs might be more representative of such heterogeneity. Finally, we cannot exclude the possibility that variability in the timing of EV sample collection across different disease stages may contribute to discrepancies in detected LAPM levels. Indeed, evidence of diurnal variation in leukemia-specific biomarkers in PB, as well as circadian fluctuations in hematologic parameters and EV levels, suggest that such effects cannot be excluded [60,61]. Future investigations should address this issue through standardized sampling protocols and larger patient cohorts.

Of note, our findings, together with previous studies, demonstrate that LAPMs are present in EVs. However, the mechanisms underlying their selective incorporation into EVs remain to be understood. While general EV cargo sorting mechanisms (e.g., ESCRT-dependent pathways, tetraspanin-enriched microdomains, and lipid raft associations) have been described for other membrane proteins, it remains to be determined whether LAPMs follow similar or distinct pathways. Further investigation into these mechanisms will be important to understand the regulation of LAPM packaging into EVs. Moreover, the potential for analytical interference from normal leukocyte-EVs is also a significant consideration in liquid biopsy research due to shared protein markers. However, although certain LAPMs are also present on normal leukocytes and their corresponding EVs, these markers typically become diagnostically meaningful when aberrantly expressed, namely through cross-lineage antigen expression, abnormal levels of expression, or asynchronous expression of immature and mature antigens [16].

To date, to our knowledge there are no peer-reviewed studies establishing EV-based MRD detection in AML. Prior studies have primarily focused on detecting LAPMs in EVs at single time points, most commonly at diagnosis; however, they have not addressed MRD detection or longitudinal dynamics across disease stages [62,63,64]. In the present study, we confirmed that clinically relevant LAPMs are present in AML-derived EVs. Moreover, by systematically comparing multiple EV isolation methods, we identified SEC-UF as the most reliable approach. We also highlight the importance of using a consistent isolation methodology for comparative longitudinal analyses, ensuring that observed changes reflect biological variation rather than technical artifacts, and acknowledge that, at present, the techniques used in this study may pose challenges for implementation and standardization in the clinical practice, an issue that warrants consideration in future work. Importantly, among EV isolation strategies, SEC represents one of the most promising approaches for clinical translation due to its relatively low cost, simplicity, reproducibility, and minimal requirement for specialized infrastructure [65,66]. In addition, SEC has been reported to preserve EV integrity, biological activity, and initial abundance, while remaining compatible with scalable and standardized workflows, making it well suited for clinical and multi-center applications [67]. Nevertheless, significant challenges remain in the downstream analysis and clinical integration of EV-based MRD assessment. In particular, while flow cytometry is a cornerstone technique for cellular MRD assessment in hematological malignancies, its application to EVs is not yet fully standardized. Accurate EV detection by flow cytometry requires dedicated high-sensitivity instrumentation capable of resolving submicron particles, such as nanoscale flow cytometers or optimized high-resolution. These instruments, although powerful, are associated with high acquisition and maintenance costs, require specialized operator training, and still lack full harmonization of analytical protocols, limiting their current widespread clinical implementation [68]. Due to its robustness and broad availability in research and clinical laboratories, in this study we use Western blot for detecting the presence of LAPMs in EV-associated proteins. In particular, experiments in the present study were designed as proof-of-concept studies, aimed at demonstrating the feasibility of detecting LAPMs in AML cell- and patient-derived EVs rather than providing definitive quantitative validation. We are aware that Western blot is inherently a semi-quantitative and low-throughput technique, which limits its applicability as a stand-alone method for routine MRD monitoring. Future advances in high-throughput and clinically adapted EV-detection platforms will be essential to bridge the gap between proof-of-concept studies and translational application.

Nonetheless, while larger studies are necessary to validate these findings, this work provides a foundational framework for the future development of EV-based real-time MRD monitoring in AML.

## 5. Conclusions

The present proof-of-concept study paved the way for the potential applicability of a PB EV-based liquid biopsy MRD monitoring approach, having demonstrated the detection of LAPMs in the cargo of AML-derived EVs. In the future, validation of these results in a larger cohort of patients will be required, and healthy controls must be included to establish thresholds of the disease burden. Indeed, the inclusion of healthy control samples in the present study would have improved the value of the presented data; however, the reported results in a cell line and further validated in AML patient samples are of value for the proof-of-concept study described in this manuscript. The significance of such proof-of concept studies is unquestionable, serving as a foundation for the execution of larger-scale studies in future work [69,70,71]. Comparison of this approach with the conventional MRD determination approach is also necessary, to truly assess the advantages of this approach over the traditional approach. In the future, we intend to validate this preliminary study by including more patients and comparing the LAPMs detected in EVs with the immunophenotypic data obtained in the clinical setting. Such comparison will be necessary to validate the usefulness of LAPMs detection in plasma EVs. Nevertheless, additional efforts to optimize and standardize the applied EV isolation methodology must be taken in order to assure its applicability into daily clinical practice.

## Figures and Tables

**Table 1:** Top 15 EV-associated protein markers detected in EVs released by OCI-AML3 cells, based on the MISEV2023 guidelines [28], presented by order of abundance. Mean of abundances normalized and coefficient of variation (CV) for each protein detected are shown.

	UniProt Accession Number	Protein	Gene Symbol	Mean Abundance (Normalized)	Abundances CV [%]
1	P04406	Glyceraldehyde-3-phosphate dehydrogenase	GAPDH	1.66 × 10^9^	68.99
2	P08238	Heat shock protein HSP 90-beta	HSP90AB1	1.31 × 10^9^	69.38
3	P11142	Heat shock cognate 71 kDa protein	HSPA8	7.74 × 10^8^	64.44
4	Q71U36	Tubulin alpha-1A chain	TUBA1A	5.28 × 10^8^	67.28
5	P07355	Annexin A2	ANXA2	3.62 × 10^8^	67.73
6	P08133	Annexin A6	ANXA6	3.47 × 10^8^	81.64
7	O43707	Alpha-actinin 4	ACTN4	2.78 × 10^8^	62.04
8	P04899	Guanine nucleotide-binding protein G(i) subunit alpha-2	GNAI2	2.55 × 10^8^	77.36
9	P35613	Basigin	BSG	2.02 × 10^8^	52.66
10	P05556	Integrin beta-1	ITGB1	1.65 × 10^8^	58.16
11	Q8WUM4	Programmed cell death 6-interacting protein	PDCD6IP	1.24 × 10^8^	59.09
12	P08575	Receptor-type tyrosine-protein phosphatase C	PTPRC	1.23 × 10^8^	89.44
13	P60033	CD81 antigen	CD81	1.05 × 10^8^	88.25
14	O00560	Syntenin-1	SDCBP	1.04 × 10^8^	73.5
15	P08648	Integrin alpha-5	ITGA5	7.37 × 10^7^	54.37

**Table 2:** List of LAPMs detected by quantitative proteomic analysis of OCI-AML3 cells and their released EVs. Mean of abundances normalized and coefficient of variation (CV) for each protein detected are shown. n/d: non-detected.

UniProt Accession Number	Protein	Gene Symbol	Mean Abundance in Cells (Normalized)	Abundances CV in Cells [%]	Mean Abundance in EVs (Normalized)	Abundances CV in EVs [%]
O95400	CD2 antigen cytoplasmic tail-binding protein 2	CD2BP2	2.70 × 10^6^	4.15	n/d	n/d
P08571	Monocyte differentiation antigen CD14	CD14	1.99 × 10^6^	41.78	4.31 × 10^5^	96.11
P20138	Myeloid cell surface antigen CD33	CD33	1.20 × 10^5^	65.19	3.47 × 10^5^	104.34

**Table 3:** Recovery volume and time required to complete the protocol for three distinct methodologies for EV isolation from AML patients’ PPP. Results for size exclusion chromatography followed by ultrafiltration, Total Exosome Isolation Kit (Invitrogen) and Exo-Spin^TM^ Exosome Blood Purification Kit (Cell Guidance Systems) are shown.

Isolation Method	Final Volume	Duration
Size Exclusion Chromatography followed by Ultrafiltration	~156 µL	3 h 30
Total Exosome Isolation Kit	200 µL	1 h 15
Exo-Spin^TM^ Exosome Blood Purification Kit	800 µL (200 µL × 4 columns)	4 h 00

## Data Availability

The original contributions presented in this study are included in the article/Supplementary Material. Further inquiries can be directed to the corresponding author(s).

## Supplementary Materials

The following supporting information can be downloaded at https://www.mdpi.com/article/10.3390/cells15121068/s1, Figure S1: Gene Ontology analysis of the proteins present in EVs released by the OCI-AML3 cell line, regarding the most enriched biological processes, namely cellular process (A) and metabolic process (B); Figure S2: Gene Ontology analysis of the proteins present in EVs released by the OCI-AML3 cell line, regarding the most molecular functions, namely binding (A) and catalytic activity (B); Table S1: top 10 most abundant proteins present in EVs shed by the OCI-AML3 cell line.

## Abbreviations

The following abbreviations are used in this manuscript:

ACTN4	Alpha-Actinin 4
AHSG	Alpha-2-HS-Glycoprotein
ALIX	Programmed Cell Death 6-Interacting Protein
AML	Acute Myeloid Leukemia
ANXA2	Annexin A2
ANXA6	Annexin A6
ATCC	American Type Culture Collection
BM	Bone Marrow
BSG	Basigin
CD2BP2	CD2 Antigen Cytoplasmic Tail-Binding Protein 2
CD14	Monocyte Differentiation Antigen CD14
CD33	Myeloid Cell Surface Antigen CD33
CHUSJ	Centro Hospitalar Universitário de São João
DMEM	Dulbecco’s Modified Eagle Medium
ENO1	Alpha-Enolase
EV	Extracellular Vesicle
FBS	Fetal Bovine Serum
FDR	False-Discovery Rate
GAPDH	Guanine Nucleotide-Binding Protein G(i) Subunit Alpha-2
HEMS	Histology and Electron Microscopy
HSPA1	Heat Shock Cognate 71 kDa Protein
HSP90AB1	Heat Shock Protein HSP 90-Beta
ITGB1	Integrin Beta-1
ITGBA5	Integrin Alpha-5
LAPMS	Leukemia-Associated Phenotypic Protein Markers
LC-MS/MS	Liquid Chromatography with Tandem Mass Spectometry
MICAL1	[F-actin]-Monooxygenase
MRD	Measurable Residual Disease
MSN	Moesin
NTA	Nanoparticle Tracking Analysis
NONO	Non-POU Domain-Containing Octamer-Binding Protein
PB	Peripheral Blood
PPP	Poor Platelet Plasma
PTPRC	Receptor-Type Tyrosine-Protein Phosphatase C
RPMI	Roswell Park Memorial Institute
SDCBP	Syntenin-1
SEC-UF	Size Exclusion Chromatography followed by Ultrafiltration
SP3	Solid-Phase-Enhanced Sample-Preparation
TBS	Tris-Buffered Saline Solution
TEM	Transmission Electron Microscopy
TUBA1A	Tubulin Alpha-1A Chain
WB	Western Blot
