# Supplementary material for: A Minimally Invasive, Extracellular Vesicle-Based Approach for Monitoring Measurable Residual Disease in Acute Myeloid Leukemia: A Proof-of-Concept Study
Source: Cells. 2026 Jun 11;15(12):1068. doi: 10.3390/cells15121068 (PMC13297537; doi:10.3390/cells15121068)
Supplement: Supplementary file 1 [file cells-15-01068-s001.zip › cells-4319655-supplementary.pdf]

Supplementary Data

A Minimally-invasive Extracellular Vesicle-based Approach for Monitoring Measurable Residual Disease in Acute Myeloid Leukemia

Helena Branco [1,2,#], Joana Carreira [1,2,3,#], Inês Soure [1,2,4], Cristina P.R. Xavier [1,2,5,6,*], Andreia Rosário [1,2], Maria Amorim [7], Hugo Osório [1,2,8], José E. Guimarães [1,2,5,7,8], Ana Bela Sarmento-Ribeiro [9,10,11], Manuel A. Sobrinho-Simões [1,2,7,8], Hugo R. Caires [1,2], M. Helena Vasconcelos [1,2,12,*]

1 i3S – Instituto de Investigação e Inovação em Saúde, Universidade do Porto, Rua Alfredo Allen 208, 4200-135 Porto, Portugal; hbranco@ipatimup.pt (H.B.); carreirajoana@hotmail.com (J.C.); ines.msoure@gmail.com (I.S.); andreia_rosario11@hotmail.com (A.R.); hosorio@i3s.up.pt (H.O.); hcaires@i3s.up.pt (H.R.C.)
2 Cancer Drug Resistance Group, IPATIMUP – Institute of Molecular Pathology and Immunology, University of Porto, Rua Júlio Amaral de Carvalho 45, 4200-135 Porto, Portugal
3 FMUC – Faculty of Medicine, University of Coimbra, Azinhaga de Santa Comba, 3000-548 Coimbra, Portugal; ana.belasarmento@gmail.com (A.B.S.-R.)
4 Department of Chemistry, University of Aveiro, Campus Universitário de Santiago, 3810-193 Aveiro, Portugal
5 Associate Laboratory i4HB – Institute for Health and Bioeconomy, University Institute of Health Sciences – CESPU, Gandra, Portugal
6 UCIBIO – Applied Molecular Biosciences Unit, Toxicologic Pathology Research Laboratory, University Institute of Health Sciences (1H-TOXRUN, IUCS-CESPU), Gandra, Portugal
7 Serviço de Hematologia Clínica, Centro Hospitalar Universitário de São João, Alameda Prof. Hernâni Monteiro, 4200-319 Porto, Portugal; amorim.ml@gmail.com (M.A.); jeteguima-raes@gmail.com (J.E.G.); manuel.simoes@chsj.min-saude.pt (M.A.S.-S)
8 FMUP – Faculty of Medicine, University of Porto, Alameda Prof. Hernâni Monteiro, 4200-319 Porto, Portugal
9 Laboratório de Oncobiologia e Hematologia, Clinica Universitária de Hematologia, FMUC – Faculty of Medicine, University of Coimbra, Azinhaga de Santa Comba, 3000-548 Coimbra, Portugal
10 Coimbra Institute for Clinical and Biomedical Research (iCBR) – Grupo de Ambiente, Genética e Oncobiologia (CIMAGO) and Center for Innovative Biomedicine and Biotechnology (CIBB), University of Coimbra, Azinhaga Santa Comba, 3000-548 Coimbra, Portugal
11 Serviço de Hematologia Clínica, Centro Hospitalar e Universitário de Coimbra (CHUC), Praceta Professor Mota Pinto, 30004-561 Coimbra, Portugal
12 Department of Biological Sciences, FFUP – Faculty of Pharmacy, University of Porto, Rua de Jorge Viterbo Ferreira 228, 4050-313 Porto, Portugal

# These authors equally contributed to this work.
*Correspondence: cristina.xavier@iucs.cespu.pt (C.P.R.X.); hvasconcelos@ipatimup.pt (M.H.V.).

Supplementary Figures

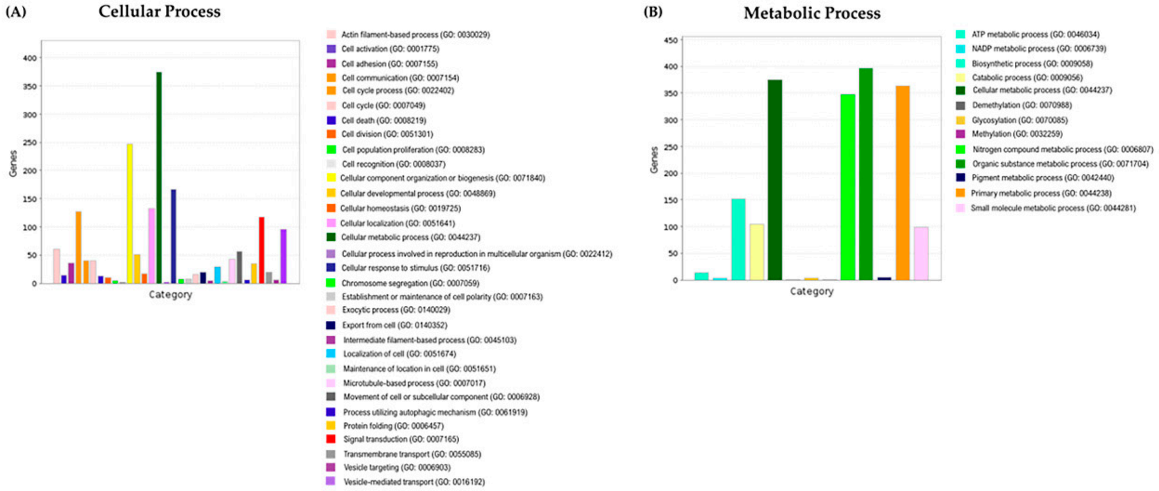

Supplementary Figure S1. Gene Ontology analysis of the proteins present in EVs released by the OCI-AML3 cell line, regarding the most enriched biological processes, namely cellular process (A) and metabolic process (B). Bar graphs were obtained through the PANTHER classification system [1].

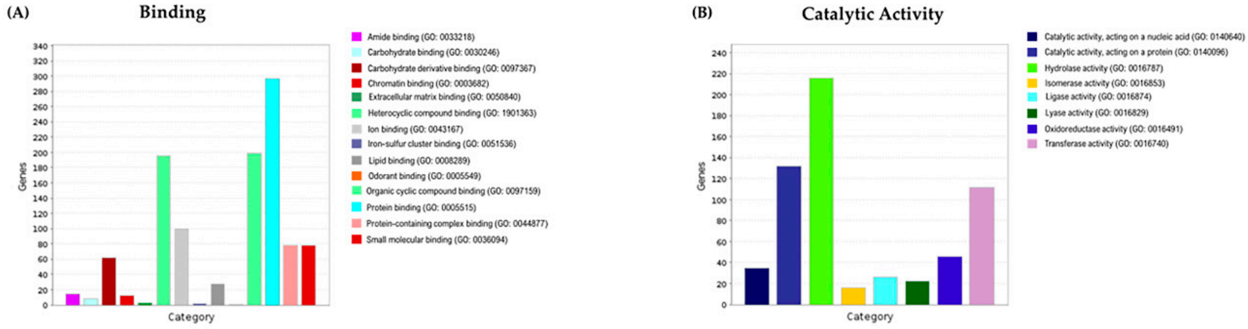

Supplementary Figure S2. Gene Ontology analysis of the proteins present in EVs released by the OCI-AML3 cell line, regarding the most enriched molecular functions, namely binding (A) and catalytic activity (B). Bar graphs were obtained through the PANTHER classification system [1].

Supplementary Tables

Supplementary Table S1. Top 10 most abundant proteins present in EVs shed by the OCI-AML3 cell line. Mean of abundances normalized and coefficient of variation (CV) for each protein detected are shown.

	UniProt Accession number	Protein	Gene symbol	Mean abundance (normalized)	Abundances CV [%]
**1**	P04406	Glyceraldehyde-3-phosphate dehydrogenase	GAPDH	1.66×10^{9}	68.99
**2**	P26038	Moesin	MSN	1.65×10^{9}	68.36
**3**	P01023	Alpha-2-macroglobulin	A2M	1.62×10^{9}	13.2
**4**	P21333	Filamin-A	FLNA	1.58×10^{9}	79.23
**5**	Q15233	Non-POU domain-containing octamer-binding protein	NONO	1.49×10^{9}	68.5
**6**	P08238	Heat shock protein HSP 90-beta	HSP90AB1	1.31×10^{9}	69.38
**7**	P06733	Alpha-enolase	ENO1	1.25×10^{9}	59.48
**8**	P02765	Alpha-2-HS-glycoprotein	AHSG	1.11×10^{9}	29.84
**9**	P35579	Myosin-9	MYH9	9.63×10^{8}	69.29
**10**	Q8TDZ2	[F-actin]-monooxygenase	MICAL1	9.33×10^{8}	83.01

References

1. Mi, H.; Muruganujan, A.; Huang, X.; Ebert, D.; Mills, C.; Guo, X.; Thomas, P.D. Protocol Update for large-scale genome and gene function analysis with the PANTHER classification system (v.14.0). *Nature protocols* **2019**, *14*, 703-721.
